# A case report of rare intraperitoneal cholesteatoma diagnosed and treated through multidisciplinary collaboration

**DOI:** 10.1002/ccr3.3075

**Published:** 2020-07-07

**Authors:** Jie Jiang, Guang Chu, Gang Chen, Hongyun Liao, Lu Yu, Hongjun Yu, Jie Liu, Zongqiang Hu

**Affiliations:** ^1^ Department of Hepatobiliary Surgery The First People's Hospital of Kunming City & Galmette Affiliated Hospital of Kunming Medical University Kunming China

**Keywords:** abdominal tumor, cholesteatoma, diffusion‐weighted imaging (DWI), multidisciplinary team (MDT), surgical treatment, teratoma

## Abstract

55‐year‐old female patient with abdominal distension and poor appetite for 3 months was diagnosed as intraperitoneal cholesteatoma by imaging findings and histological tests. Patient has received surgical resection and recovered well after operation.

## Graphical abstract

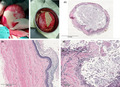

## 1 INTRODUCTION OF CASE DATA

Here, we report a rare case of intraperitoneal cholesteatoma. Magnetic resonance imaging showed a giant mass lesion in the right abdominal cavity of patient, which was considered more likely to be a “cholesteatoma” from the perspective of imaging. The pathological diagnosis after surgical resection was intraperitoneal cholesteatoma.

**FIGURE 1:**
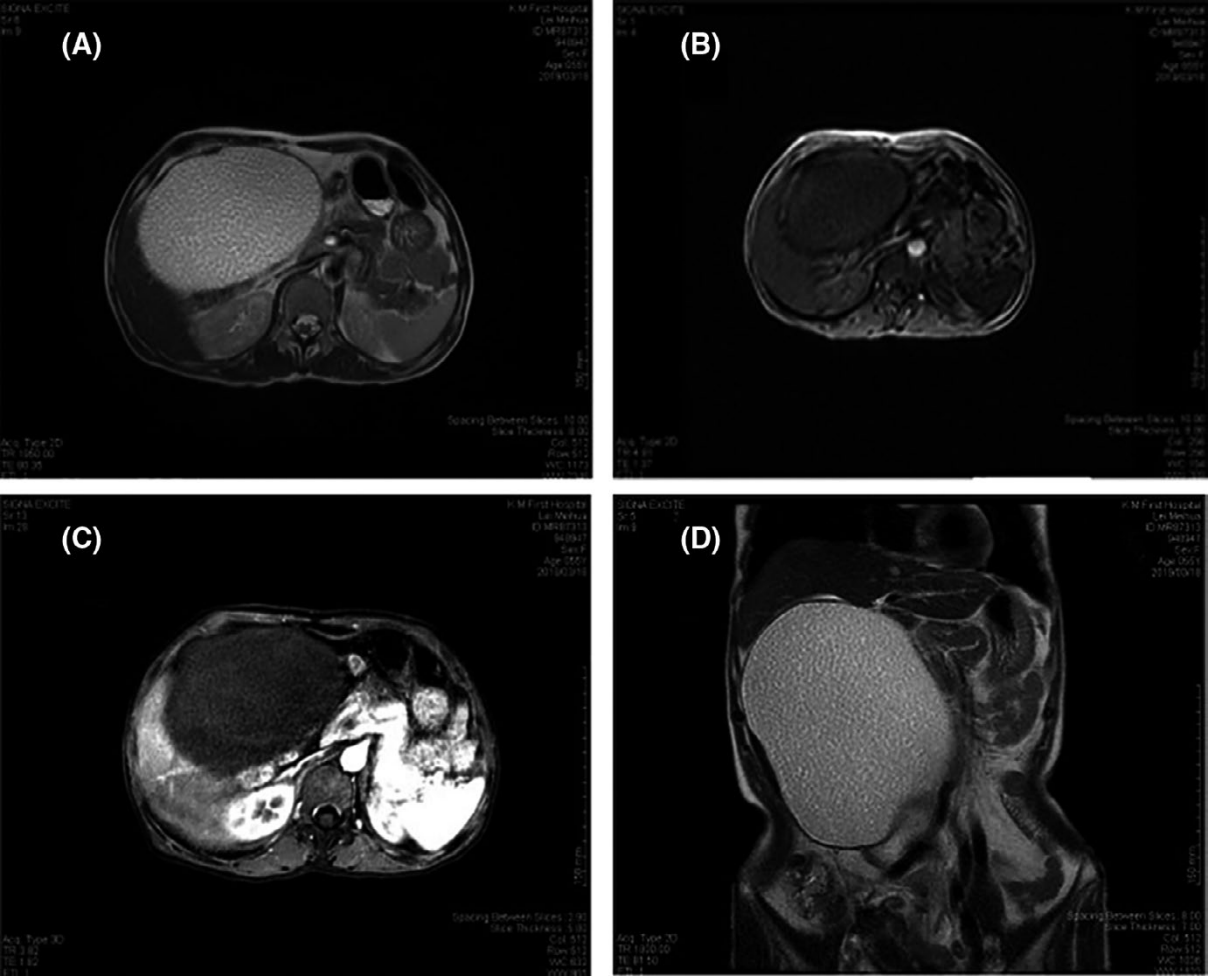
MRI results of the patient. A, T1WI: giant mass‐like high signal shadow with clear boundaries. B, T1 inversion: low signal shadow with clear boundaries. C, Enhancement: no obvious enhancement. D, T2WI coronal view: high signal shadow with obvious compression of adjacent tissues. Summary: A large mass with long T1 and long T2 signals was observed in the right abdominal cavity, with a smooth edge and clear boundaries; the size was approximately 10.3 × 14.2 × 18.3 cm. There was no significant enhancement at the edge in enhanced imaging

Cholesteatoma is a kind of benign tumor formed by abnormal squamous epithelial keratosis, most cases occurred in the middle ear, mastoid process, cerebellopontine angle, suprasellar cistern, skull, and temporal bone, while intraperitoneal cholesteatoma in very rare. Here, we report a rare case of intraperitoneal cholesteatoma. The patient (female, 55 years old) was admitted to the hospital because of abdominal distension and poor appetite for more than 3 months. Approximately 4 months before admission, the patient went to the local hospital for health examination, the ultrasound examination showed a huge cyst (10 × 12 cm) in the abdominal cavity, and further examination and treatment were recommended. However, the patient had no abdominal pain, abdominal distension or other discomfort at that time, and she did not seek further examination or treatment. One month later, the patient felt obvious abdominal distension, which was aggravated after eating. Subsequently, the patient developed poor appetite and fatigue, without nausea and vomiting or other discomfort. In the recent 3 months, the patient's body weight did not change significantly, and there was no abnormality in urination and defecation. The patient had been postmenopausal for 5 years and was not sexually active. Physical examination after admission revealed that the entire abdomen was slightly distended and more pronounced on the right side. A mass approximately 8 × 10 cm in size was palpable on the right side of the abdomen with a clear border, smooth surface, no tenderness, and poor mobility. Abdominal MRI showed a giant mass lesion (10.3 × 14.2 × 18.3 cm) in the right abdominal cavity, which had a smooth edge and clear border, with hypointense on T1‐weighted images and hyperintense on T2‐weighted (see Figure 1A,B,D). 3D volume‐enhanced imaging showed no significant intense enhancement at the edge of the lesion (see Figure 1C). The organs and large blood vessels around the mass were displaced by squeezing. The volume of the liver was significantly increased. In the liver near the dome of the diaphragm, inside and outside the left lateral lobe, inside the right posterior lobe, and in the space between the spleen and stomach, there were multiple different sized circle‐like lesions with hypointense on T1‐weighted images and hyperintense on T2‐weighted images, which were similar to signals of abdominal space‐occupying lesions. Based on the above findings, multiple intraperitoneal cholesteatoma was suspected. Blood culture, blood gas analysis, routine coagulation tests, parasite screening, immunological screening, cardiopulmonary function, and human chorionic gonadotropin (HCG) showed no significant abnormalities. Diagnosis on admission was intraperitoneal space‐occupying lesion (nature to be determined: cholesteatoma?). Other laboratory test results are shown in Table 1.

**Table 1:** Laboratory Data

	Reference range	At admission	2 d after surgery	4 d after surgery	7 d after surgery
Tumor marker					
CEA (ng/mL)	<3.4	11.9			
CA199 (IU/mL)	<39	185.5			
Liver function					
TP (g/L)	60.0‐87.0	72.0	46.7	57.8	59.7
ALB (g/L)	35.0‐56.0	39.2	23.5	33.2	35.1
A/G	1.50‐2.50	1.20	1.01	1.35	1.43
ALT (IU/L)	30‐65	21.0	30.0	33.0	28.0
AST (IU/L)	<40	27.0	46.0	36.0	21.0
ALP (IU/L)	30‐100	28.0	15.0	12.0	14.0
K (mmol/L)	3.5‐5.5	3.63	3.29	2.42	3.09
Ca (mmol/L)	2.0‐2.7	2.20	1.90	2.01	2.11
MG (mmol/L)	0.8‐1.2	0.75	0.39	0.60	0.65
P (mmol/L)	1.0‐1.6	1.14	0.82	0.90	1.01
Hs‐CPR (mg/L)	0.0‐3.0	1.30	51.5	40.1	7.90
Routine blood tests					
HGB (g/L)	110‐150	118	97.0	102	103
RBC (10^12^/L)	3.5‐5.0	3.62	2.93	3.12	3.13
WBC (10^9^/L)	4.0‐10.0	5.76	11.63	8.88	6.30
NEUT %	50.0‐70.0	43.1	79.1	76.7	48.3
LYNM %	20.0‐40.0	48.4	14.5	17.6	38.1
CK (IU/L)	20‐220		763		

Note

Reference values are affected by many variables, including the patient population and the laboratory methods used. The ranges used in the First Hospital of Kunming are for adults who are not pregnant and do not have medical conditions that could affect the results. They may therefore not be appropriate for all patients.

## 2 DIFFERENTIAL DIAGNOSIS

1. Hepatic cystic lesions: Hepatic cystic lesions can be classified as infectious or noninfectious lesions.
  1. Noninfectious cystic lesions of the liver are further divided into benign, precancerous, malignant, and traumatic lesions, among which Hepatic simple cysts are more common. Some patients can be diagnosed by ultrasound because of the clinical symptoms of cyst compression, such as abdominal pain, early satiety, nausea, and vomiting. The sensitivity and specificity of ultrasound in the diagnosis of simple cysts are about 90%. Some patients need to be examined by CT or MRI to determine their size and nature. Differential diagnosis is especially important. Differential diagnosis is highly important.^1^, ^2^
  2. Among infectious cystic lesions of the liver, parasitic lesions such as hydatid cysts, diagnosis is based in part on clinical history. Attention should be paid to the patient's place of residence, place of origin, and occupation to identify high‐risk patients. The most common symptom is pain in the upper right quadrant or upper abdomen accompanied by nonspecific symptoms, such as nausea, fever, dyspepsia, or allergy.^3^ Rupture of hydatid cysts can cause fever, pruritus, eosinophilia, or fatal anaphylaxis responses. These cysts can be identified by parasite‐specific antibody screening, CT and MRI.^4^

- Benign and malignant solid tumors of the liver: Most liver tumors are malignant, and cavernous hemangiomas and liver cysts are the most common primary benign liver tumors and tumor‐like lesions. At present, abdominal ultrasonography, CT, and MRI are the most commonly used imaging examination for the solid tumors of the liver due to its ease of use. Malignant liver tumors are often associated with the medical history of the patient, such as hepatitis B and alcohol consumption.^5^
- Benign ovarian tumors: Benign ovarian tumors account for approximately 25.0%‐33.3% of benign female genital tumors, which include serous adenomas, teratomas, and ovarian corpus luteum cysts. Early clinical symptoms are not obvious. As the condition progresses, the patient may develop an abdominal mass, abdominal pain, abdominal distension, menstrual disorder, prolonged menstrual period, infertility, and other symptoms. B ultrasound, MRI, and serum tumor markers facilitate diagnosis.^6^
- Ovarian cancer: Ovarian cancer is a kind of common female genital malignancies, of which the etiology is not clear. In addition, ovarian cancer is the most malignant tumor of the female reproductive system. Its onset is insidious, and most of the clinical symptoms occurred in the period of advanced cancer. Symptoms such as abdominal mass, ascites, and abdominal pain occur, and metastases are widespread in the pelvic and abdominal cavities. The mortality is very high. Gynecological examination, tumor markers, MRI, etc, can facilitate differential diagnosis, and pathological examination can make a definite diagnosis.^7^

## 3 DIAGNOSIS, TREATMENT, AND REVIEW

Combined with the existing examination results, the nature of the abdominal space‐occupying lesion could not yet be determined. However, the symptoms of patient such as abdominal distension and anorexia are further aggravated, which indicates that the abdominal cavity space‐occupying lesion and oppresses the surrounding organs and tissues seriously. After discussion, an exploratory laparotomy was planned to relieve the compression and confirm the diagnosis.

**FIGURE 2:**
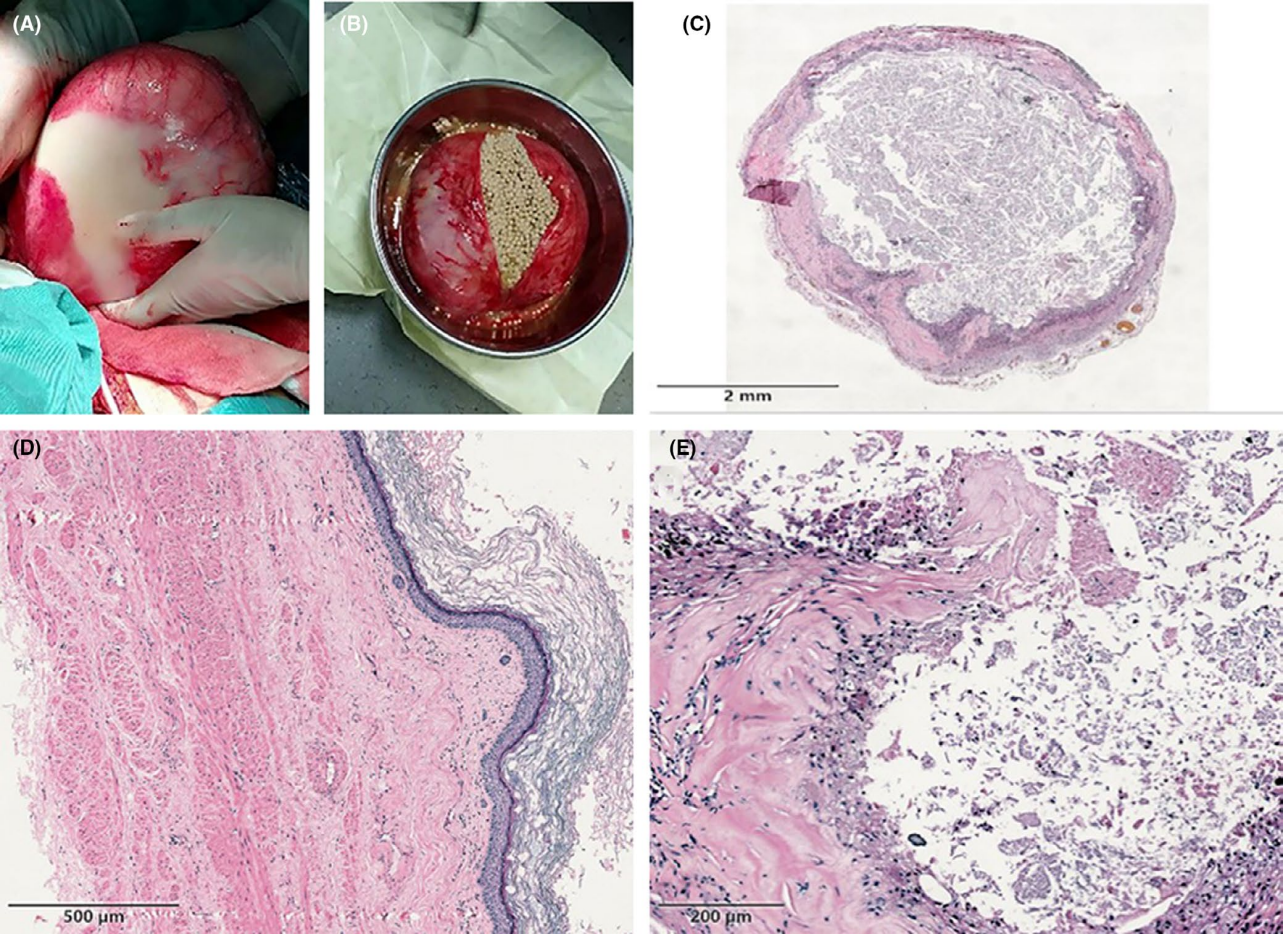
Pathological examination results of the mass. A, Dissection of the surgical specimen showed a cyst with a size of 15 × 15 × 10 cm, of which the capsule was complete with high tension. The surface showed local yellowish‐white changes. The mass was yellowish‐white, and the texture was tough and hard. The length of the oviduct tissue was 12.5 cm, and its diameter was 0.8 cm. B, The thickness of the cyst wall was 0.2‐1.5 cm, and inside the cyst, there were several grayish white nodules with diameters of 0.1‐0.7 cm and a large amount of fluid (approximately 2000 ml). C, Pathological examination results showed “pearly tumors” with sizes of approximately 4‐6 mm. The surface was covered with mesangium, which contained tiny blood vessels. See Figure e for the tissues inside the mesangium. D, The outermost cyst wall tissue, in which blood vessels and skin appendages were visible. E, The structure under the mesangium of the “pearly tumor”: stratified squamous epithelial cells, in which keratinized protein was found

After performing the relevant preoperative examinations within 1 week of admission, exploratory laparotomy was performed under general anesthesia. Along the 25 cm right paramedian abdominal incision, the abdominal mass (15 × 15 × 10 cm, see Figure 2A) was fully exposed after layer‐by‐layer laparotomy. The capsule of the mass was intact with high tension. The surface of the mass showed local yellowish‐white changes, and the mass was tough, with a poor blood supply. The explored mass originated from the right ovary and had no adhesion with the surrounding organs. There were several ectopic proliferative masses (several masses were on the greater omentum or free, one mass was on the diaphragmatic surface of the spleen, two masses were on the diaphragmatic surface of the liver, and one mass was on the right posterior lobe of liver). Bilateral adnexectomy + hepatosplenic ectopic hyperplasia resection + enterolysis were performed, and all masses detected in the abdominal cavity were completely removed. Intraoperative specimen dissection was performed, and numerous lesions with “pearl‐like” tumor characteristics contained light yellowish fluid (see Figure 2B). Histological examinations showed “pearly tumors” with sizes of approximately 4‐6 mm. The surface was covered with mesangium, which contained tiny blood vessels (see Figure 2C). The pathological examination also revealed that the cyst wall was covered with stratified squamous epithelium, and a keratinized substance was visible within the cyst (see Figure 2D,E).

CT was performed 7 days after the operation. The liver changed after the operation; the punctate high‐density shadow visible in the upper segment of the right lobe of the liver was no longer present, and the patchy low‐density shadow visible in the liver had been resected. The cystic low‐density shadow visible in the right abdominal cavity had been resected. The spleen showed postoperative changes. The multiple ring shadows visible in the intestinal canal of the right lower abdomen were no longer visible, and the bilateral adnexa had been removed.

## 4 DISCUSSION

Cholesteatoma is a benign tumor, which is an enlarged collection of keratins in a squamous epithelial sac. Because the tumor contains abundant keratins, cholesterol, lipids, calcification and other substances, the appearance of the cyst wall is thin, white and lustrous, and translucent, and the cyst has a pearl‐like shape with a clear boundary. Cholesteatomas do not have an abundant blood supply and have various sizes; they are known as “pearly tumors”.^8^, ^9^ Due to the different ratios of squamous epithelial debris and keratinized protein in the cyst, the lesion can cystic or pseudo‐solid, which results from the etiology (congenital or acquired).^1^ In addition, most cases occur in the middle ear, mastoid process, cerebellopontine angle, suprasellar cistern, skull and temporal bone and other parts of the body ^10^; intraperitoneal cholesteatoma is very rare and rarely reported in the literature in China and abroad.

Under MRI, cholesteatoma is mainly shown as hypointense on T1‐weighted images and hyperintense on T2‐weighted images without significant enhancement, and diffusion‐weighted imaging (DWI) is an effective method for differentiating cholesteatoma from other cystic lesions.^11^, ^12^, ^13^ A study by Williams et al found that cholesteatoma is avascular and cannot be enhanced with contrast agent in MRI,^14^ whereas granulation tissue is poorly vascularized and is enhanced on delayed imaging. Studies by Schwartz et al found that cholesteatoma showed high intensity in DWI because of limited water fusion and partly because its keratin content produced high signal intensity in the T2 penetrating effect area of the diseased tissue and that the high signal intensity of cholesteatoma in DWI images had a higher diagnostic accuracy.^15^, ^16^ In the diagnosis of primary, residual, or recurrent cholesteatoma, the assessment of DWI is objective and reliable. In recent years, studies by Atsushi Fukuda and other scholars found that a high concentration of protein,^17^, ^18^ not cholesterol and triglyceride content, is the cause of high signal intensity in T1‐weighted images of cholesteatoma.

According to pathology, whether the cholesteatoma is congenital or acquired, they manifest as cystic, variably sized, white to pearly masses containing creamy or waxy material. In the histological diagnosis, when stratified keratinized squamous epithelium, subepithelial fibrous connective tissue or granulation tissue, and keratin debris are observed by microscopy, it can be concluded that the cyst content is composed of exfoliated epithelial cells and cholesterol crystals.^19^, ^20^, ^21^

## 5 FINAL DIAGNOSIS

Multiple intraperitoneal cholesteatoma, ovarian cystic teratoma (cholesteatoma).

## CONFLICT OF INTEREST

None declared.

## AUTHOR CONTRIBUTION

All authors: contributed to editing and revision of this manuscript. ZH: conceived the idea for the manuscript and managed the patient, and also revised the manuscript. JJ and GChu: involved in the diagnosis and treatment of the patient. JJ: wrote the main parts of the manuscript. GChen and HL: did a literature search and summarized the laboratory data in table. LY: provided the MRI images and wrote the discussion of MRI results of the patient. HY and JL: provided the pathology images and edited the pathology section. All authors: read and approved the final version of the manuscript.
